# Predictive value of soluble CD40L combined with APACHE II score in elderly patients with sepsis in the emergency department

**DOI:** 10.1186/s12871-023-02381-w

**Published:** 2024-01-19

**Authors:** Long Yang, Jun Yang, Xiangqun Zhang, Xinghua Ye, Yugeng Liu, Bing Wei, Junyu Wang

**Affiliations:** Emergency Medicine Clinical Research Center, Beijing Chao-Yang Hospital, Capital Medical University, & Beijing Key Laboratory of Cardiopulmonary Cerebral Resuscitation. Clinical Center for Medicine in Acute Infection, Capital Medical University, Beijing, 100020 China

**Keywords:** sCD40L, APACHE II score, 28-day mortality

## Abstract

**Background:**

The prognostic performance of soluble CD40L (sCD40L) for illness severity in infectious diseases is rarely reported. We investigated the ability of sCD40L combined with Acute Physiology and Chronic Health Evaluation II (APACHE II) score to evaluate mortality in septic patients in the emergency department(ED).

**Methods:**

We enrolled 222 septic patients in the ED of Beijing Chao-Yang Hospital from October 2020 to April 2021. Their serum sCD40L, PCT, lactate (Lac), Sequential Organ Failure Assessment (SOFA) score, Acute Physiology and Chronic Health Evaluation II (APACHE II) score were used to predict the prognosis of septic patients in terms of 28-day mortality. Serum sCD40L was detected by Human XL Cytokine Luminex. Logistic regression analysis and receiver operating characteristic (ROC) curves were used to assess the prognostic value of the variables.

**Results:**

One hundred ninety-five patients met the inclusion criteria, divided into survival group (55 cases) and non-survival group (140 cases). sCD40L, PCT, Lac, SOFA and APACHE II score were found to independently predict 28-day mortality (*P* < 0.05). The AUC values of sCD40L, PCT, Lac, SOFA and APACHE II score were 0.662,0.727,0.704, 0.719 and 0.716, respectively. There was no difference in the diagnostic value of sCD40L compared with the PCT, Lac, SOFA score or APACHE II score (*Z*_1_ = 1.19, *P* = 0.234; *Z*_2_ = 0.77, *P* = 0.441; *Z*_3_ = 1.05, *P* = 0.294; *Z*_4_ = 0.97, *P* = 0.332). However, the combined evaluation of sCD40L + APACHE II (AUC:0.772, *Z* = 2.10, *P* = 0.036) was much better than sCD40L alone in predicting 28-day mortality.

**Conclusion:**

The predictive value of sCD40L + APACHE II is better than sCD40L alone for 28-day mortality. sCD40L combined with APACHE II score is valuable for predicting 28-day mortality in elderly patients with sepsis.

## Background

Sepsis is an organ dysfunction syndrome caused by the body’s dysfunctional response to infection, mainly manifested as chills, fever (or hypothermia), palpitation, shortness of breath, mental state changes and other symptoms [1]. Septic patients are often critically ill and their condition seems to progress quickly, leaving little time for doctors to react, and that’s why early and prompt recognition and reasonable treatment can significantly reduce mortality [2]. With the rapid development of severe infection medical practices, many existing biomarkers can no longer meet the current clinical needs, such as the peak CRP levels, according to the latest research, did not independently predict mortality in patients with bacteremia in the ED [3] and new and more predictors need to be explored.

CD40L(CD154), a 39-kD transmembrane glycoprotein associated with tumor necrosis factor-α (TNF-α), was originally identified on stimulated CD4-positive T cells, mast cells, and basophils [4, 5]. CD40L has a variety of pro-inflammatory and pro-coagulant effects, including inflammation caused by interleukin-1, TNF-α, and tissue factors, as well as upregulation of endothelial cell adhesion molecules [6]. CD40L stimulates resting platelet activity by binding constitutionally expressed CD40 during cell contact [7]. When platelets are activated, they quickly carry CD40L to the cell surface [6]. Activated platelets release a large number of inflammatory mediators [8–10]. After activation and expression on the cell membrane surface, platelets release CD40L in a solute form (sCD40L) [11]. sCD40L as a platelet agonist is related to *α* II b *β* 3 ligand properties [12]. Platelet-derived sCD40L is involved in stimulating neutrophil activation and Mac-1(macrophage-1) expression, as well as promoting neutrophil lung infiltration and sepsis associated lung injury [13]. A few studies have found that the relationship between sCD40L and sepsis, and proved that the elevated serum sCD40L was associated with poor prognosis [14–17].

Hence, our study focuses on confirming whether sCD40L combined with APACHE II score can positively impact the mechanisms of prediction and have a satisfactory effect on the prognosis of patients with sepsis.

## Methods

### Patients and grouping

The study was a single-center observational study conducted in the emergency department of Beijing Chao-Yang Hospital. From October 2020 to April 2021, 222 consecutive septic patients according to Sepsis-3 (SOFA score ≥ 2) [18] were enrolled in our cohort. The exclusion criteria were as follows: age younger than 18 years, end-stage disease (malignant tumor with metastases, AIDS, end-stage kidney or liver disease), as well as patients who refused to participate in the study or who had some missing or incomplete data.

### Data collection

Blood samples were taken on emergency admission. White blood cell count, blood biochemistry, blood gas and serum sCD40L levels were detected within 24 hours after admission. PCT, CRP, Lac, ALB(albumin), AST (aspartate aminotransferase), ALT (alanine transaminase) and Scr (serum creatinine) were tested in the lab. PCT was determined by Bio merieux Mini VIDAS immunoassay. Serum sCD40L concentration was detected by using Human XL Cytokine Luminex® Performance Assay 46-plex Fixed Panel (LKTM014B, R&D). The reference range of the level of sCD40L for predicting 28-day mortality is about 1.37 ng/ml to 6.07 ng/ml [13]. Each sample was measured in duplicate. The SOFA and APACHE II scores were calculated when patients arrived at the emergency department. All patients with sepsis were followed up for 28 days. The 28-day mortality was the end point of the study.

### Statistical analysis

All data were analyzed using SPSS version 24.0 (SPSS Inc., Chicago, IL, USA). Data were expressed as mean ± standard deviation or median, and for normally distributed continuous variables, an independent sample T-test was used for comparison. The Mann-Whitney *U* test was used to compare the two groups of distributed data. The frequency was compared by Chi-square test. Multiple logistic regression analysis was used to determine the independent outcome predictors. Receiver operating characteristic (ROC) curves and the area under the curves (AUC) were conducted to compare the predictive value. The Kaplan-Meier curve was used to describe the cumulative survival rate. All statistical tests were 2-tailed, and *P* < 0.05 was considered statistically significant [19]. The combination of variables was realized through the ROC curve of SPSS software, which was a commonly used statistical method for sepsis research. The following logistic regression equation was developed based on the β-coefficient of the sCD40L and other variables: L = sCD40L + β1/β2*variable, which L is the combination value of sCD40L and other variables [20].

## Results

### Basic information and characteristics of the cohort

**Fig. 1:**
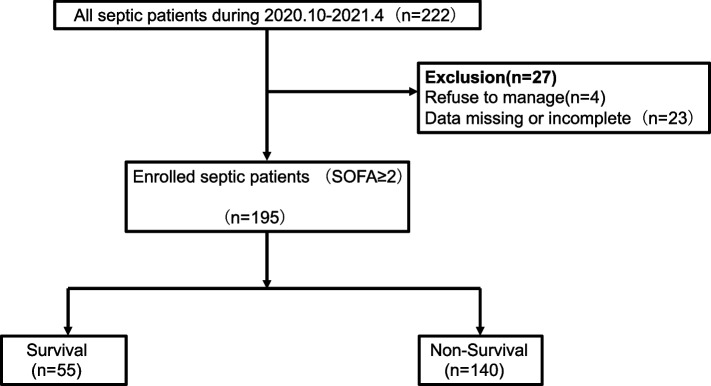
Basic information of the study cohort

From October 2020 to April 2021, 222 patients with sepsis were screened consecutively. We excluded 27 patients declined to participate or with data missing or incomplete, and thus included 195 participants divided into survival group (55 cases) and non-survival group (140 cases), completed the 28-day follow-up (Fig. 1).

The median age of survival group was higher than non-survival group (71 vs 70, *P* < 0.05). The Median level of Lac, CRP, SOFA score, APACHE II score and sCD40L were much higher in non-survival group than in survival group(*P* < 0.05). The gender, COPD (chronic obstructive pulmonary disease), hypertension, DM (diabetes mellitus), CVD (cerebrovascular disease), CHF (congestive heart failure), WBC, AST and ALT were not different between survival group and non-survival group (Table 1).

**Table 1:** Basic information of the study cohort

Variables	Survivor(*n* = 55)	Non-Survivors(*n = 140*)	*P*
Age, years	71.05 ± 14.89	70.75 ± 14.81	0.001
Male, %	32 (58.2)	81 (58.0)	0.967
COPD, %	9(16.4)	31 (22.1)	0.368
Hypertension, %	26(47.2)	61(43,6)	0.640
CVD, %	9(16.4)	26(18.6)	0.718
CHF, %	20(36.4)	71(50.7)	0.071
DM, %	15(27.3)	43(30.7)	0.636
MAP, mmHg	98(86,108)	93 (83,103)	0.040
Temperature,°C	36.4 ± 0.6	36.4 ± 2.9	0.001
HR, beats/min	94(76,113)	86(75,101)	0.088
RR, bpm	20(18,22)	20(19,23)	0.035
WBC, *10^9/L	8.7(7.1,13.2)	9.3(7.0,11.6)	0.700
PLT, *10^9/L	201(152,302)	176(136,251)	0.075
Lac, mmol/L	1.2(1.0, 1.5)	1.5(1.1, 2.3)	0.002
Scr mmol/L	72.5(53.2, 84.3)	64.1(48.5, 84.9)	0.196
ALB g/L	34.8 ± 8.2	32.7 ± 8.6	0.001
AST U/L	23.8(18.2, 32.5)	28.3(19.3, 43.3)	0.064
ALT U/L	20.3(16.4,25.9)	22.2(15.6,31.9)	0.376
PCT(ng/ml)	0.05(0.05,0.07)	0.05(0.05,1.39)	0.004
CRP(mg/L)	8(5,35)	20(8,80)	0.013
sCD40L (pg/ml)	2194 (1750, 2922)	2520 (1779, 4203)	0.043
SOFA	6(5,8)	7(5,10)	0.022
APACHE II	16(13,22)	24(17,33)	0.001

Abbreviation: *sCD40L* soluble CD40L, *WBC* white blood cell, *PCT* procalcitonin, *Lac* lactate, *CRP* C-reactive protein, *ALB* albumin, *Scr* serum creatinine, *AST* aspartate aminotransferase, *ALT* alanine transaminase, *PLT* platelet, *MAP* mean arterial pressure, *RR* respiratory rate, *HR* heart rate, *SOFA* Sequential Organ Failure Assessment, *APACHE II* Acute Physiology and Chronic Health Evaluation II, *COPD* chronic obstructive pulmonary disease, *CVD* cerebrovascular disease, *CHF* congestive heart failure, *DM* diabetes mellitus

**Fig. 2:**
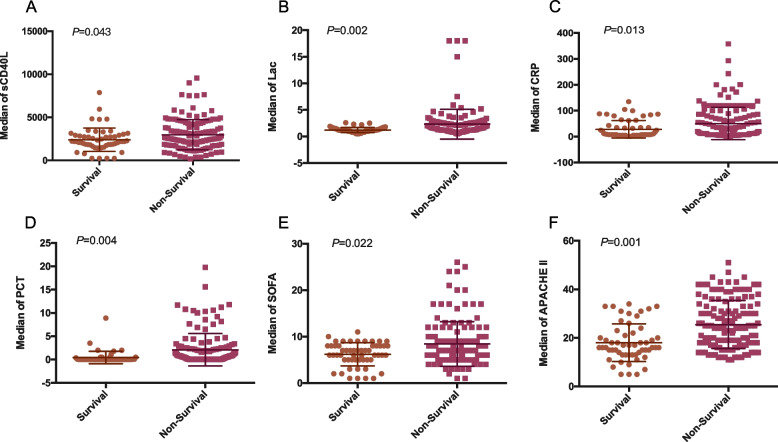
The median levels of sCD40L, Lac, CRP, PCT, SOFA score and APACHE II score in 28-day mortality groups(**A-F**)

The median of the sCD40L, Lac, PCT, CRP, SOFA and APACHE II scores are shown in Fig. 2. There were significant differences in the average levels of sCD40L, Lac, CRP and PCT, as well as SOFA or APACHE II score between survival and non-survival patients(*P* < 0.05).

### Spearman’s correlations of sCD40L with other indicators

**Fig. 3:**
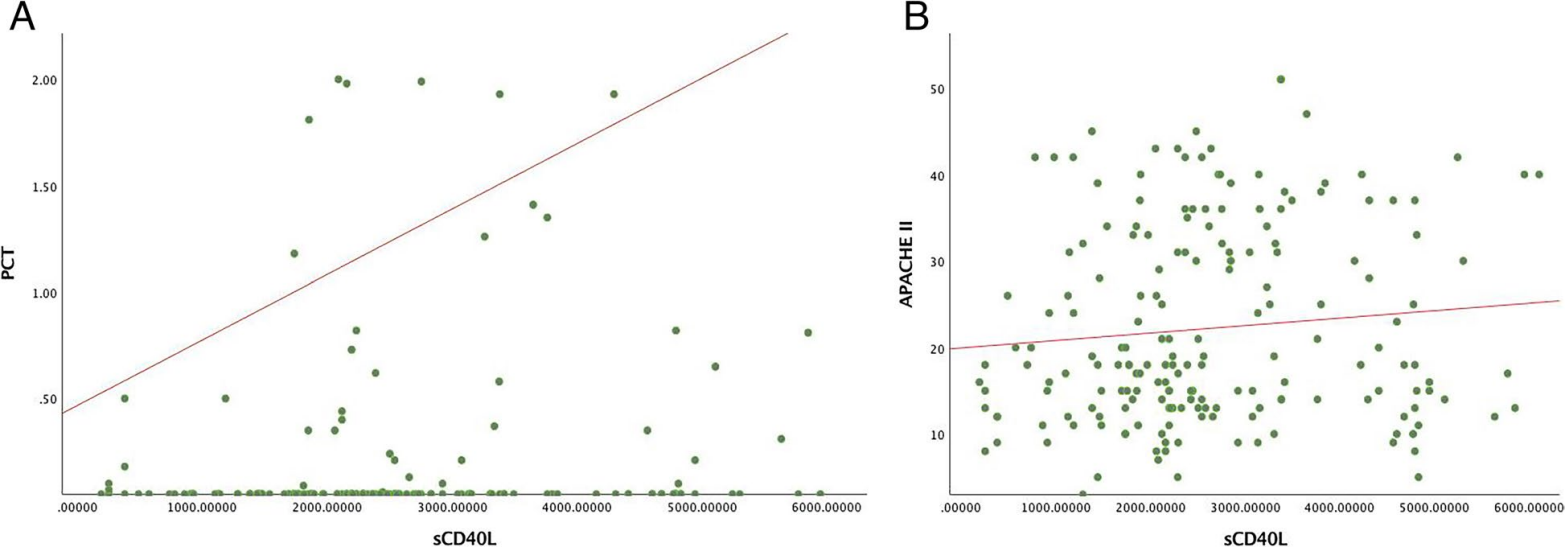
Spearman’s correlations of septic patients. sCD40L with PCT (**A**); sCD40L with APACHE II score (**B**)

To investigate the correlations between sCD40L and WBC, PLT, AST, ALT, Lac, CRP, PCT, SOFA score and APACHE II score, Spearman’s correlation analysis was conducted (Table 2). Figure 3A, B showed the positive liner correlations of sCD40L with PCT (*r =* 0.183, *P <* 0.05) and sCD40L with APACHE II score (*r* = 0.148, *P <* 0.05).

**Table 2:** Spearman’s correlations analysis between sCD40L and other indicators

Variables	WBC	PLT	AST	ALT	Lac	CRP	PCT	SOFA	APACHE II
Spearman correlation	0.087	−0.055	0.034	−0.079	0.120	0.033	0.183	0.066	0.148
*P*	0.226	0.444	0.639	0.271	0.095	0.649	0.010	0.358	0.040

Abbreviation: *sCD40L* soluble CD40L, *WBC* white blood cell, *PCT* procalcitonin, *Lac* lactate, *CRP* C-reactive protein, *AST* aspartate aminotransferase, *ALT* alanine transaminase, *PLT* platelet, *SOFA* Sequential Organ Failure Assessment, *APACHE II* Acute Physiology and Chronic Health Evaluation II

### Logistic regression analysis of the predictive value of septic patients

We used the multivariate logistic regression to analyze independent predictors for this study. The results presented that sCD40L, Lac, PCT, SOFA and APACHE II score were the independent predictors of 28-day mortality, but neither WBC nor CRP (Table 3).

**Table 3:** Multivariate logistic regression analysis of 28-day mortality for septic patients

Variables	*β*	*SE*	Wald	*P*	OR(95%CI)
sCD40L	0.001	0.001	4.246	0.039	1.000 (1.000–1.001)
WBC	0.023	0.045	0.257	0.612	1.023 (0.936–1.119)
Lac	0.714	0.287	6.216	0.013	2.043 (1.165–3.583)
CRP	0.008	0.005	2.592	0.107	1.008 (0.998–1.018)
PCT	0.264	0.134	3.906	0.048	1.302 (1.002–1.692)
SOFA	0.291	0.081	12.804	0.001	1.338 (1.141–1.570)
APACHE II	−0.086	0.024	12.330	0.001	0.918 (0.875–0.963)
Constant	−0.408	0.679	0.361	0.548	

Abbreviation: *sCD40L* soluble CD40L, *WBC* white blood cell, *PCT* procalcitonin, *Lac* lactate, *CRP* C-reactive protein, *SOFA* Sequential Organ Failure Assessment, *APACHE II* Acute Physiology and Chronic Health Evaluation II

The following logistic regression equation was developed based on the β-coefficient of the sCD40L,SOFA,APACHE II, Lac and PCT:

$$\textrm{L}1=\textrm{sCD}40\textrm{L}+\frac{\ 0.291}{0.001}\ast \textrm{SOFA},$$

$$\textrm{L}2=\textrm{sCD}40\textrm{L}+\left(-\frac{0.086}{0.001}\right)\ast \textrm{APACHE II},$$

$$\textrm{L}3=\textrm{sCD}40\textrm{L}+\frac{\ 0.714}{0.001}\ast \textrm{Lac},$$

$$\textrm{L}4=\textrm{sCD}40\textrm{L}+\frac{\ 0.264}{0.001}\ast \textrm{PCT}.$$

Which L is the combination value of sCD40L and other variables [20].

### Predictive value of clinical prognosis in patients with sepsis

**Fig. 4:**
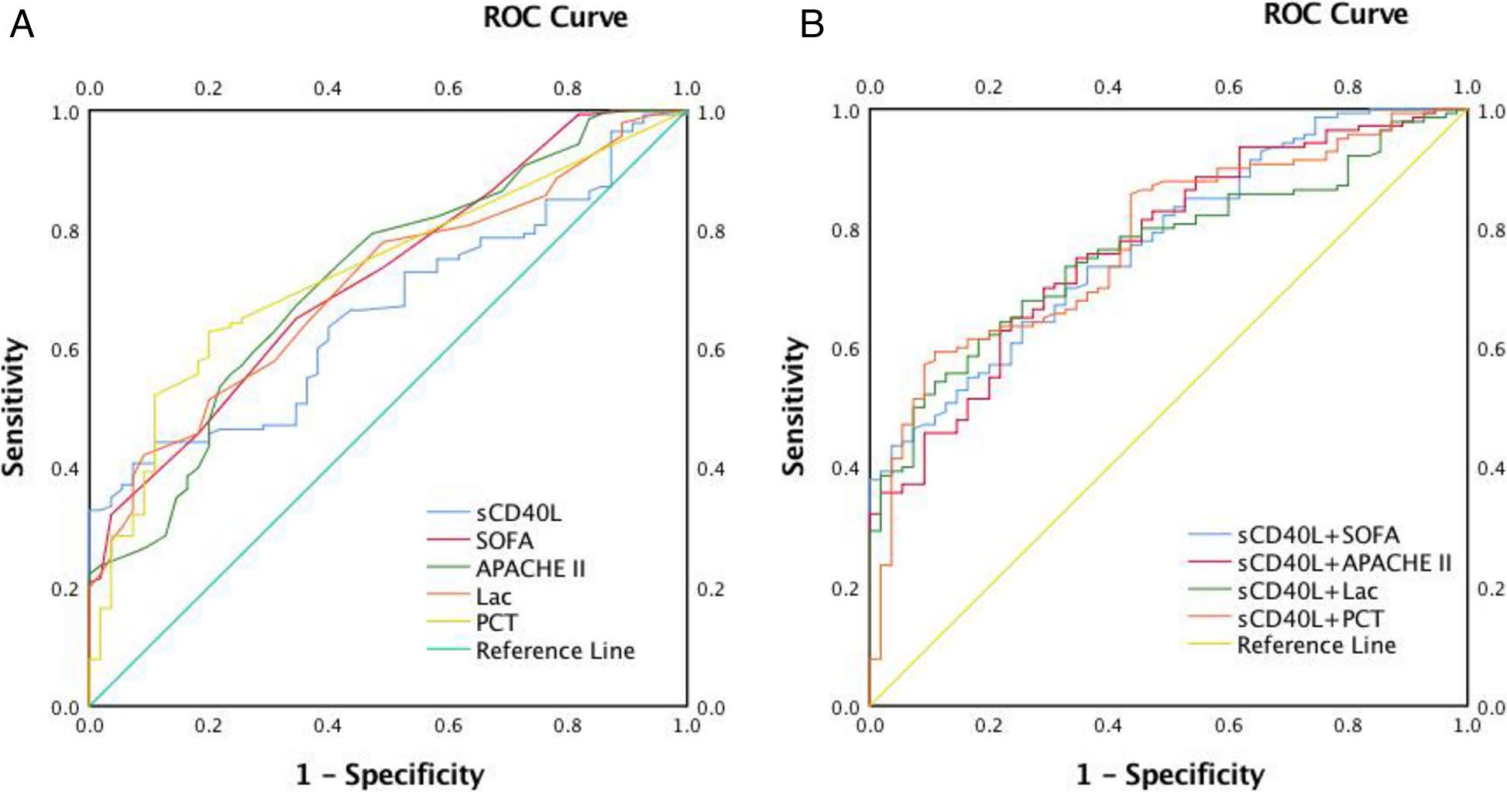
The ROC curves of variables (**A**), the ROC curves of combinations of sCD40L and other variables (**B**) for predicting 28-day mortality in patients with sepsis

The ROC curves of sCD40L and the combination of all variables to predict the 28-day mortality of patients (Fig. 4A, B and Table 4). The AUCs of the sCD40L, Lac, PCT, SOFA and APACHE II scores for 28-day mortality were 0.662, 0.727, 0.704, 0.719 and 0.716, respectively. The combination values of variables for predicting 28-day mortality were as follows: sCD40L + SOFA: 0.775; sCD40L + APACHE II: 0.772; sCD40L + Lac: 0.762; sCD40L + PCT: 0.778. Statistically, there was no difference in the diagnostic value of sCD40L compared with the PCT, Lac, SOFA score or APACHE II score (*Z*_1_ = 1.19, *P* = 0.234; *Z*_2_ = 0.77, *P* = 0.441; *Z*_3_ = 1.05, *P* = 0.294; *Z*_4_ = 0.97, *P* = 0.332). However, sCD40L + APACHE II score (AUC:0.772, *Z* = 2.10, *P* = 0.036) was much better than sCD40L alone in predicting 28-day mortality.

**Table 4:** Statistical data of ROC curve in predicting 28-day-mortality in septic patients

Variables	AUC (95%CI)	*SE*	*P*	Cut off	Sensitivity	Specificity
sCD40L	0.662 (0.586–0.739)	0.039	< 0.001	3060.31	0.443	0.891
SOFA	0.719 (0.643–0.794)	0.038	< 0.001	7.5	0.650	0.655
APACHE II	0.716 (0.638–0.794)	0.040	< 0.001	18.5	0.671	0.655
Lac	0.704 (0.629–0.779)	0.038	< 0.001	1.9	0.421	0.909
PCT	0.727 (0.653–0.801)	0.038	< 0.001	0.12	0.629	0.800
sCD40L + SOFA	0.775 (0.708–0.842)	0.034	< 0.001	0.85	0.436	0.964
sCD40L + APACHEII	0.772 (0.703–0.840)	0.035	< 0.001	0.71	0.650	0.764
sCD40L + Lac	0.762 (0.695–0.829)	0.034	< 0.001	0.79	0.543	0.891
sCD40L + PCT	0.778 (0.710–0.846)	0.035	< 0.001	0.74	0.593	0.891

Abbreviation: *sCD40L* soluble CD40L, *PCT* procalcitonin, *Lac* lactate, *SOFA* Sequential Organ Failure Assessment, *APACHE II* Acute Physiology and Chronic Health Evaluation II

### The cumulative 28-day mortality of sepsis

**Fig. 5:**
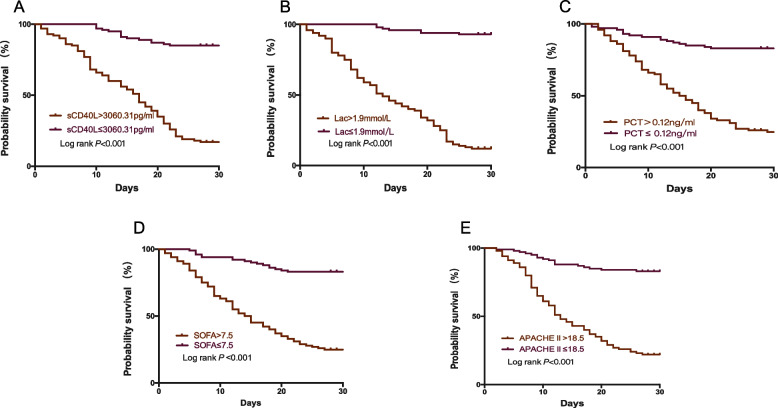
The cumulative 28-day survival rate of variables in patients with sepsis*.* sCD40L(**A**), Lac (**B**), PCT(**C**), SOFA score(**D**), and APACHE II score(**E**)

**Fig. 6:**
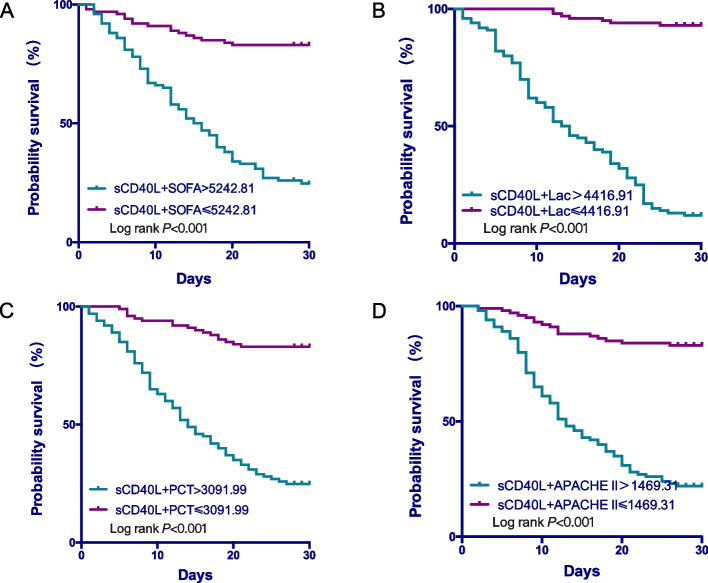
The cumulative 28-day survival rate of combinations of sCD40L and other variables in patients with sepsis*.* sCD40L + SOFA (**A**), sCD40L + Lac (**B**), sCD40L + PCT(**C**), sCD40L+ APACHE II (**D**)

The cumulative 28-day survival was lower in septic patients with sCD40L > 3060.31 pg/ml, Lac> 1.9 mmol/L, PCT > 0.12 ng/ml, SOFA score > 7.5 and APACHE II score > 18.5, respectively (Fig. 5). When comparing the combined diagnostic value of sCD40L with other factors, sCD40L + SOFA> 5242.81, sCD40L + APACHE II > 1469.31, sCD40L + Lac> 4416.91, sCD40L + PCT > 3091.99 showed a higher mortality rate (Fig. 6).

## Discussion

The main finding of this study was that the predictive value of sCD40L + APACHE II score was better than sCD40L alone for 28-day mortality. sCD40L combined with APACHE II score had a higher predictive value for 28-day mortality in elderly patients with sepsis.

Our study found that serum sCD40L levels in the non-survival group were higher than those in the survival group, in agreement with previous studies [14–17]. Furthermore, serum sCD40L levels>3060.31 pg/ml were found associated with higher 28-day mortality by multiple logistic regression.

We considered age, temperature, respiratory rate, Lac, ALB, PCT, CRP, sCD40L, APACHE II score, and SOFA score as risk factors associated with mortality. Our outcomes showed that other factors, such as chronic diseases, WBC, Scr, AST, and ALT were not statistically different between survival and non-survival groups. We also found that some septic diagnosis variables were associated with 28-day mortality, such as sCD40L, Lac, PCT, APACHE II score and SOFA score.

It is likely that the pro-inflammatory and pro-thrombotic effects of serum sCD40L could increase the risk of death in patients with sepsis [21]. Platelet-derived mediators may be involved in the process of granulomatous chronic inflammation [22]. Activated platelets release a large number of inflammatory mediators [8–10]. Platelets are activated in damaged tissues, and certain foreign substances enter the body to activate platelets [23]. In the tumor necrosis factor superfamily, CD40L is involved in the occurrence of chronic inflammation associated with granuloma. CD40L-CD40, is involved in a variety of immunomodulatory and inflammation-related responses [24, 25]. CD40 induces macrophages to express cytokines, tissue factors, and matrix metalloproteinases (MMPs) [26–28]. CD40-CD40L interactions stimulates vascular endothelial cells and macrophages to induce adhesion and molecules [29, 30], generation of tissue factor(TF) [31, 32] and release of pro-inflammatory cytokines [33, 34], which participate in inflammatory response and thrombosis formation. CD40L could up-regulate TF and thrombomodulin [35, 36], as well as E-selection and vascular cell adhesion molecule-1(VCAM-1)independently of TNF-α or IL-1β. CD40L, which is shed from platelets or expressed on their surfaces, can activate endothelial cells(ECs), causing their activity to change to promote inflammation or thrombosis. After the expression of platelet cell membrane, CD40L is cleaved by specific enzymes into the sCD40L and released into the blood to function. sCD40L is mediated by GPIIb/IIIa, a surface glycoprotein that binds fibrinogen, and by MMPs [37]. All above effects can promote inflammation, endothelial thrombosis, organ dysfunction and even death.

Our study did not confirm any associations between serum sCD40L levels and platelet counts, although approximately 95% of sCD40L was derived from platelets [37], which was different from previous results presented by other scholars [14]. Traditionally, in most of the infections, platelet counts are often reduced, a phenomenon easily identifiable in patients with sepsis. Differently, sCD40L levels were considerably elevated in patients with sepsis, which can be considered a contradictory outcome. Although our study did not confirm the existence of a positive correlation between sCD40L levels and platelet count, our results are still of great significance and the shortcomings might be related to our insufficient sample size or lack of focus on plasma sCD40L. Our study was limited to serum sCD40L due to the previous reports that confirmed the levels of sCD40L in serum was significantly higher than that in plasma [38]. In a future follow-up study, we would like to expand the clinical sample size and continue to deepen the research on this topic.

We also found that sCD40L levels were positively associated with criteria of sepsis severity, such as APACHE II scores and PCT. In clinical diagnosis and treatment, the APACHE II score and PCT are commonly used to evaluate the severity of the disease. The higher the APACHE II score, the more critical the condition is, and the worse the prognosis, which is likely to imply higher risks of death. Similarly, the higher the PCT, the more severe the bacterial infection, which worsens the risk of developing severe disease and the overall clinical prognosis. Our results showed that sCD40L levels were significantly higher in the non-survival group than in the survival group and independently predicted 28-day mortality in septic patients. These outcomes are in good agreement with the results presented by Leonardo et al. [14]. In critical disease evaluation, sCD40L, PCT, and APACHE II score all have high predictive value. As a new biomarker, sCD40L, combined with PCT or APACHE II score has a greater predictive value and is of solid significance in critical disease evaluation.

Several limitations of our study were as follows: First, our study was a single-center study with a small sample size, requiring more centers and a larger number of samples to join the cohort; Second, this research only measured sCD40L levels in serum and not in plasma samples, and further study should be paid more attention to sCD40L levels both in serum and plasmas samples; Third, we determined sCD40L levels only 24 hours after admission, not one week or more, this might influence the results of the study, and further stratified studies on sample collection time would be carried out. Fourth, much more severe scores such as Mortality in Emergency Department Sepsis (MEDS) score and Predisposition, Infection, Response, and Organ dysfunction (PIRO) score should be added to our study to evaluate the severity of sepsis. There were also some positive aspects of our study, which was significant because it was the first time that sCD40L and APACHE II score had been successfully combined to assess the severity and clinical prognosis of septic patients. Meanwhile, the predictive value of sCD40L in sepsis was further proved.

## Conclusion

This study suggests that the predictive value of sCD40L + APACHE II score is better than sCD40L alone for 28-day mortality. sCD40L combined with APACHE II score has a higher predictive value for 28-day mortality in elderly patients with sepsis.

## Data Availability

All data generated or analyzed in this study are included in this paper.

## Abbreviations

sCD40L: soluble CD40L
WBC: white blood cell
PCT: procalcitonin
Lac: lactate
CRP: C-reactive protein
ALB: albumin
Scr: serum creatinine
AST: aspartate aminotransferase
ALT: alanine transaminase
PLT: platelet
MAP: mean arterial pressure
RR: respiratory rate
HR: heart rate
TNF-α: tumor necrosis factor-α
Mac-1: macrophages-1
MMP: matrix metalloproteinase
ECs: endothelial cells
TF: tissue factor
VCAM-1: adhesion molecule-1
SOFA: Sequential Organ Failure Assessment
APACHE II: Acute Physiology and Chronic Health Evaluation II
MEDS: Mortality in emergency department sepsis
PIRO: Predisposition, Infection, Response, and Organ dysfunction
COPD: chronic obstructive pulmonary disease
CVD: cerebrovascular disease
CHF: congestive heart failure
DM: diabetes mellitus
